# Comprehensive Protocol for Handling Human Small Airway Epithelial Cells (HSAECs) to Establish Air–Liquid Interface (ALI) Cultures With TEER-Based Barrier Integrity Assessment

**DOI:** 10.21769/BioProtoc.5699

**Published:** 2026-06-05

**Authors:** Dominika Jakubczyk, Marcelina Pyclik, Dominika Kozakiewicz, Józefa Macała, Agnieszka Zabłocka, Sabina Górska

**Affiliations:** Laboratory of Microbiome Immunobiology, Hirszfeld Institute of Immunology and Experimental Therapy, Polish Academy of Sciences, Wrocław, Poland

**Keywords:** Air–liquid interface (ALI), Primary cell culture, Human small airway epithelial cells (HSAECs), Transepithelial electrical resistance (TEER), Basic protocol

## Abstract

Understanding epithelial barrier function is essential for studying both its normal physiology and its role in disease, yet choosing an appropriate experimental model remains challenging. Animal models are commonly used but often suffer from interspecies differences that limit translational relevance. Human-derived cell lines offer a more suitable alternative, although establishing them often requires immortalisation strategies that involve overexpression of oncogenes, which can introduce phenotypic and functional changes. In contrast, primary cells, such as human small airway epithelial cells (HSAECs), provide a more physiologically accurate model. A critical aspect of replicating the native respiratory environment is maintaining continuous air exposure, which can be achieved through air–liquid interface (ALI) culture. This protocol provides a unified, step-by-step workflow for cultivating primary HSAECs under ALI conditions, covering the entire process from initial recovery after cryopreservation to the formation of a barrier-like layer. The protocol incorporates non-invasive methods such as transepithelial electrical resistance (TEER) measurements to monitor its integrity. While individual elements of this workflow have been described separately in different studies, a consolidated version encompassing the full workflow has not been widely available. This resource is intended for researchers with limited experience in airway epithelial culture and offers practical, clear guidance through each step of the process.

Key features

• Using primary HSAECs enables modelling the human respiratory barrier while avoiding limitations of immortalised or animal-derived cell lines.

• ALI culture technique allows continuous air exposure, closely resembling in vivo conditions for airway epithelial cells.

• TEER measurement offers a non-invasive, rapid method to assess epithelial barrier integrity without damaging the cultured cell layer.

• Protocol supports barrier function studies, including but not limited to respiratory infections, allergic responses, toxicology screening, microbiome interactions, and drug delivery investigation.

## Graphical overview


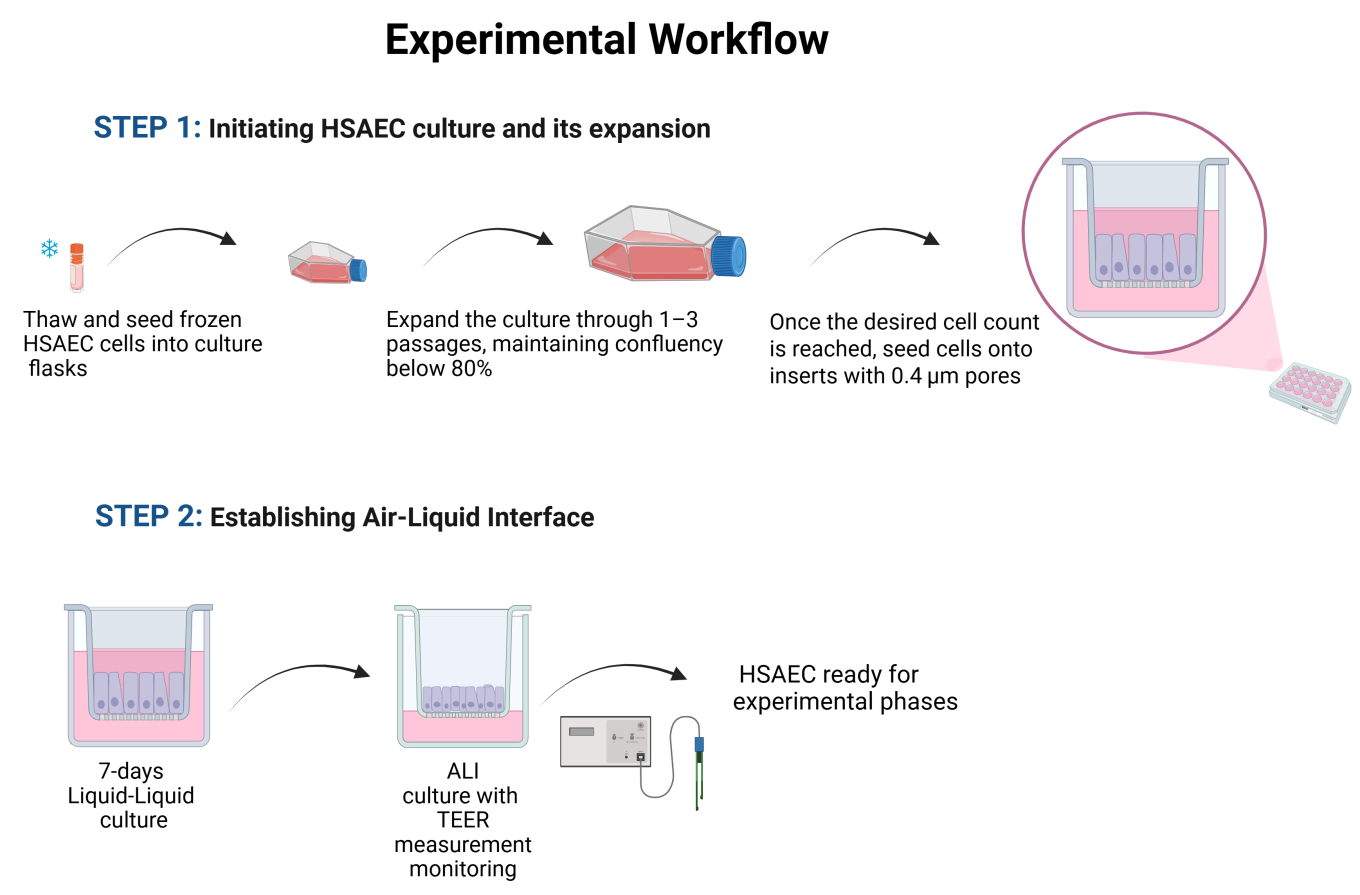


## Background

As the initial point of contact between external and internal environments, the epithelial barrier of the respiratory tract is essential for maintaining homeostasis. Humans inhale approximately 6 L of air per minute [1], with particle composition varying by location and reaching, for example, 1,300–1,900 particles per litre indoors [2]. Inhaled air may contain pollutants, viruses, bacteria, fungi, pollen, and other agents [3], which can significantly impact the integrity of the respiratory epithelial barrier and compromise health. Therefore, mapping the structure and understanding the function of the epithelial barrier are essential in studies of barrier physiology and pathophysiology, providing a foundation for exploring underlying mechanisms and evaluating therapeutic strategies. However, choosing an appropriate experimental system for such studies remains challenging. Animal models provide valuable biological insights but often exhibit interspecies differences that limit translational relevance. Immortalised human cell lines are easy to maintain, yet their establishment frequently involves genetic modifications that may alter phenotype and function. Therefore, primary human airway epithelial cells are increasingly used as an alternative model, as they retain many of the physiological characteristics necessary to reproduce the native epithelial behaviour in vitro.

Generally, primary cells are collected with the donor’s consent from specific body regions (e.g., nasal swab, bronchoscopy, intraoperative collection) and undergo minimal manipulation, thereby preserving their physiological relevance and making them suitable for studying native epithelial responses. One advantage of using primary cells is that they retain donor-specific biological diversity, allowing researchers to capture aspects of interindividual variability that are lost in immortalised systems. However, like most primary cultures, they are characterised by a limited lifespan and sensitivity to storage and handling conditions, which may influence their functional properties [4,5].

A characteristic feature of the respiratory epithelium is its continuous exposure to air, which shapes its structure, function, and responsiveness to external stimuli. When cultured under appropriate conditions, primary airway epithelial cells can form cohesive layers that reproduce essential traits, including barrier formation, allowing the examination of key aspects of epithelial physiology and pathology in a controlled setting. To support barrier development under in vitro conditions, culture systems that enable apical air contact have become fundamental tools in airway epithelial research. These approaches facilitate the formation of morphologically and functionally mature epithelial layers, providing a physiologically relevant environment for studying epithelial behaviour.

The structural integrity of such cultures can be evaluated using transepithelial electrical resistance (TEER), a non-invasive measurement of paracellular permeability [6]. A progressive increase and subsequent stabilisation of TEER values typically indicate maturation of the epithelial barrier, whereas reduced values reflect weakened tight-junction organisation. Fluctuations observed during differentiation may arise from biological changes in junctional architecture or cell morphology [7], but can also reflect the methodological differences, including electrode design, measurement conditions, calibration, or handling during assessment.

The protocol presented in this manuscript integrates these elements into a unified workflow for establishing primary cell culture and an airway-exposed model and assessing barrier integrity. While similar procedures have been reported independently across studies, consolidated, practical guidance spanning the entire process is less common. By outlining each stage in a clear, accessible manner, this protocol is intended to support researchers, particularly those with limited prior cell culture experience, in implementing reproducible, physiologically relevant airway epithelial models.

## Materials and reagents

**Biological materials**

1. Primary small airway epithelial cells (HSAECs), normal, human, derived from the lung tissue of a healthy 16-year-old Hispanic/Latino male (ATCC, catalog number: PCS-301-010)

**Reagents**

1. Airway epithelial cell basal medium (ATCC, catalog number: PCS-300-030)

2. Bronchial Epithelial Growth kit (ATCC, catalog number: PCS-300-040)

3. Penicillin-streptomycin solution (Sigma-Aldrich, catalog number: P4333)

4. Dulbecco’s phosphate-buffered saline (D-PBS) (ATCC, catalog number: 30-2200)

5. Trypsin-EDTA for primary cells containing 0.05% trypsin and 0.02% EDTA (ATCC, catalog number: PCS-999-003)

6. Trypsin neutralizing solution (ATCC, catalog number: PCS-999-004)

7. Trypan Blue 0.4% (Invitrogen, catalog number: T10282)

8. Ethanol 96% (POCH, catalog number: 396420113)

9. Ultrapure sterile water (Sigma-Aldrich, catalog number: W3500)

**Solutions**

1. HSAEC culture medium (see Recipes)

2. 70% ethanol (see Recipes)

**Recipes**

**1. HSAEC culture medium**

Transfer the indicated volume of each Bronchial Epithelial Growth kit component directly to the airway epithelial cell basal medium according to the manufacturer’s procedure:

**Table:** 

Reagent	Final concentration	Quantity or volume
HLL supplement	HSA 500 μg/mL Linoleic acid 0.6 μM Lecithin 0.6 μg/mL	1.25 mL
L-glutamine	6 mM	15 mL
Airway epithelial cell supplement	Epinephrine 1.0 μM Transferrin 5 μg/mL T3 10 nM Hydrocortisone 5 μg/mL, rh EGF 5 ng/mL rh insulin 0.1 μg/mL	5.0 mL
Optional penicillin-streptomycin solution	Penicillin: 10 Units/mL Streptomycin: 10 μg/mL	0.5 mL
Airway epithelial cell basal medium	n/a	485 mL
Total	n/a	506.75 mL

Ensure the medium is formulated under sterile conditions using a biological safety cabinet. Due to light sensitivity, the complete growth medium must be kept in the dark at 2–8 °C and should not be frozen. Storage duration should not exceed 30 days. During the procedures, it is recommended to transfer the necessary volume of medium into a separate sterile container, e.g., a 50 mL centrifuge tube, and heat only the portion required.

**2. Ethanol 70%**

**Table:** 

Reagent	Final concentration	Quantity or volume
Ethanol 96%	70%	72.9 mL
Ultrapure sterile water		27.1 mL
Total	70%	100 mL

**Laboratory supplies**

1. Tissue culture flasks, depending on the number of cells and culture stage: T25 (TPP, catalog number: 90026), T75 (TPP, catalog number: 90076), T150 (TPP, catalog number: 90151), and T300 (TPP, catalog number: 90301)

2. Dual-chamber cell counting slides (Bio-Rad, catalog number: 1450011)

3. Tissue culture test plate 24 well (TPP, catalog number: 92024)

4. ThinCerts-TC inserts 24 well, pore size 0.4 μm, transparent (Greiner Bio-One, catalog number: 662641)

5. Serological pipettes: 5 mL (GoogLab Scientific, catalog number: G33260011), 10 mL (GoogLab Scientific, catalog number: G33270011)

6. Pipetting tips: 2–100 μL (Biosphere Filter Tips, catalog number: 70.760.212), 1,000 μL (GenoPlast Biotech S.A., catalog number: GBFT100-R-NS)

7. Centrifuge tubes: 50 mL (GoogLab Scientific, catalog number: G66020522), 15 mL (GoogLab Scientific, catalog number: G66010522)

8. Eppendorf tubes 1.5 mL (Eppendorf, catalog number: EP0030120086)

## Equipment

1. Cell culture incubator with controlled temperature (37 °C), CO_2_ (5%), oxygen (20%), and humidity (90%) (Memmert, model: ICO-150MED, catalog number: ICO-150MED)

2. Biosafety cabinet class II (Thermo Scientific, model: SAFE2020, catalog number: 51026637)

3. Inverted microscope (Olympus, model: CK2, catalog number: OLY-CK2)

4. Centrifuge (Eppendorf, model: 5804R, catalog number: 5805000010)

5. Automated cell counter (Bio-Rad, model: TC20, catalog number: 1450102)

6. Epithelial Volt-Ohm Meter Millicell (Millipore, model: ERS-2, catalog number: MERS00002)

7. Adjustable automatic pipettes: 2–20 μL (HTL Lab Solution, model: Discovery Comfort, catalog number: DV20), 20–200 μL (HTL Lab Solution, model: Discovery Comfort, catalog number DV200), 100–1,000 μL (HTL Lab Solution, model: Discovery Comfort, catalog number DV1000)

8. Automatic pipettor for serological pipettes (Finetech Research and Innovation Corporation, catalog number: WIZ-EP)

9. Water bath (BioSab, model: WB-4MS, catalog number: BS-010406-AAA)

10. Sterile tweezers (Sigma-Aldrich, catalog number 930229)

11. Plastic box, e.g., Tubby container with lid (Merck, catalog number: Z675946)

## Software and datasets

1. GraphPad Prism (GraphPad Prism, Version 10), https://www.graphpad.com/scientific-software/prism/, commercial license required

2. Microsoft Excel (Microsoft Corporation, Microsoft 365 Version), https://www.microsoft.com/en-us/microsoft-365/excel, commercial license required

*Note: The software listed above were used in this protocol; however, other tools capable of performing descriptive statistical analysis and data visualisation may also be suitable, e.g., LibreOffice Calc (The Document Foundation, Version 7.6)*, *https://www.libreoffice.org/* , *free and open-source.*

## Procedure

**A. Initiating HSAEC culture from a preserved sample**

1. Prepare materials and reagents

a. Check the vial label for the number of cells in the backup sample.

b. Choose an appropriate culture flask based on a seeding density (5,000–10,000 cells/cm^2^).

c. Calculate the required volume of complete growth medium, using 3–5 mL per 25 cm^2^ of flask surface area.

d. Transfer the calculated volume of complete medium into a sterile tube and warm it to 37 °C in a water bath.

*Note: Always work under sterile conditions. Vials, tubes, and culture flasks must never be opened outside the safety cabinet. Prior to placing them inside, lightly mist their surfaces with 70% ethanol to minimise the risk of contamination. The medium warming time depends on the volume, e.g., 50 mL may take 20–30 min.*

2. Thaw the frozen cells by gently agitating the cryovial in a 37 °C water bath for 1–2 min until the content is fully thawed.

*Note: Be aware that extending the cell thawing time can negatively impact cell viability.*

3. Transfer and mix: Using a sterile pipette, transfer the thawed cell suspension into the prewarmed medium. Mix gently to ensure homogeneity.

*Note: Freshly thawed primary cells should not be centrifuged, as this may affect their viability and alter their ability to adhere.*

4. Seed the cells

a. Transfer the entire volume of cell suspension into the culture flask.

b. Incubate at 37 °C in a humidified atmosphere with 5% CO_2_ and 20% O_2_ for 24 h.

5. Assess cell attachment: After 24 h, inspect the flask under a microscope to evaluate cell adherence. If most cells are attached, proceed to medium replacement. If not, extend the incubation for another 12–14 h.

6. Replace the medium

a. Prewarm fresh medium to 37 °C.

b. Carefully aspirate the medium using a serological pipette, avoiding disturbance of attached cells, and replace with fresh medium.

7. Continue the culture with confluency monitoring

a. Return the flask to the incubator and maintain the standard culture conditions as previously described.

b. After 24–48 h, assess cell confluency under a microscope. If confluency is <70%, continue replacing the medium every 48 h. If confluency reaches 70%–80%, proceed with cell passaging.

**Figure 1.**
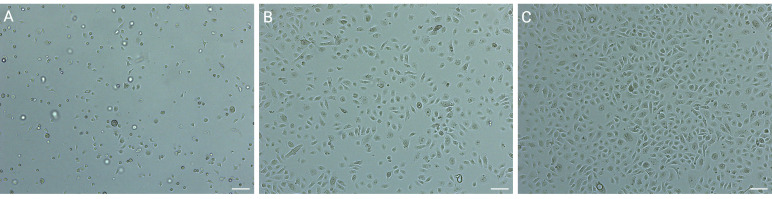
Confluency of human small airway epithelial cells (HSAECs) during the population expansion phase (4× magnification). (A) At seeding. (B) At 70%–80% confluency. (C) At >90% confluency. Scale bars, 100 μm.

*Note: HSAECs belong to contact-inhibited cells. Do not allow confluency to exceed 80%, as this may suppress proliferation and induce morphological changes that could compromise experimental outcomes. Figure 1 presents the confluence percentage of HSAEC cells in the culture flask.*

**B. HSAEC passage**

1. Prepare materials and reagents. Determine the appropriate volume of culture medium needed based on the flask size, and warm it to 37 °C before use.

*Note: Since temperature enhances enzymatic activity and facilitates cell detachment, warm both the trypsin-EDTA solution and the trypsin neutralizing solution in a 37 °C water bath. Allow the D-PBS to reach room temperature.*

2. Remove culture medium and rinse the flask bottom

a. Carefully aspirate the culture medium, taking care not to disturb the attached cells.

b. Rinse the cell layer with 3–5 mL of D-PBS to remove residual medium, then aspirate completely.

3. Apply trypsin-EDTA and monitor detachment

a. Add 2 mL of prewarmed trypsin-EDTA per 25 cm^2^ of surface area.

b. Gently rock the flask to ensure an even distribution across the cell layer.

c. Place the flask in a 37 °C incubator for 1–3 min.

d. Then, observe the detached cells under a microscope; detached cells become round and float in the solution.

*Note: If some cells remain attached, vigorously tap the bottom of the flask from multiple sides to facilitate detachment. Do not use any cell scraper as it damages the cells and reduces the number of viable cells. If needed, return the flask to the incubator for an additional 1–2 min. Do not exceed a total incubation time of 5 min, as prolonged exposure to trypsin can damage cells. Overexposure to trypsin may cause white streaks to appear in the solution; if this occurs, promptly neutralise the solution.*

4. Neutralise trypsin and collect detached cells

a. Once most cells are detached, add trypsin neutralising solution in an equal volume to the trypsin.

b. Gently pipette to ensure thorough mixing and complete neutralisation.

c. Transfer the cell suspension to a sterile centrifuge tube.

d. To recover remaining cells, rinse the flask bottom 2–3 times with 5 mL of D-PBS and combine all the fractions with the original suspension.

5. Centrifuge and resuspend the obtained pellet in fresh medium

a. Centrifuge the cell suspension at 150× *g* for 5 min to pellet the cells.

b. Carefully aspirate the supernatant and then resuspend the cell pellet in 1–3 mL of fresh, prewarmed complete growth medium, adjusting the volume based on the pellet size.

**Figure 2.**
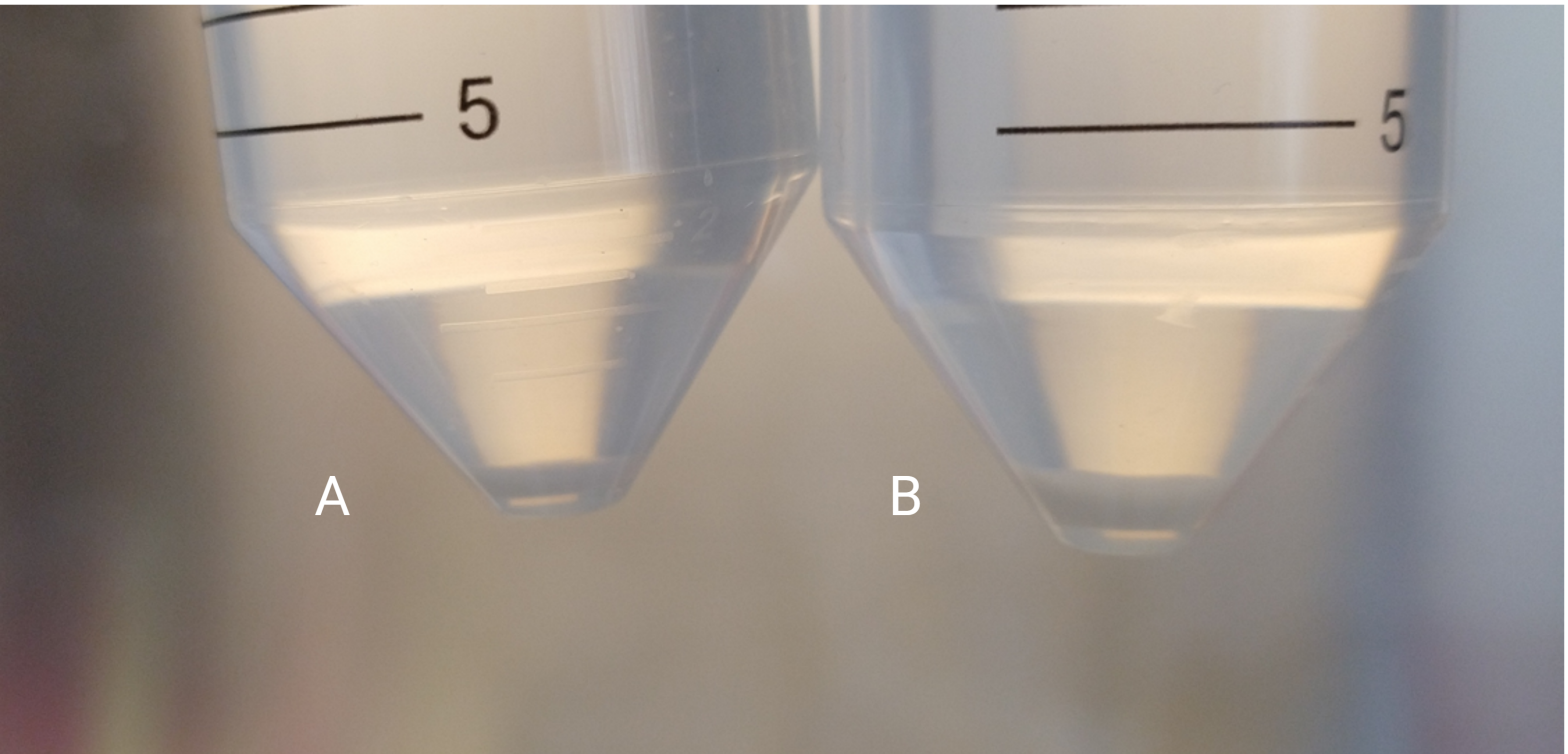
Representative cell pellet sizes after centrifugation. (A) Smaller pellet size; recommended 1 mL of medium for resuspension. (B) More compact pellet; recommended 2–3 mL of medium for resuspension.

*Note: It is important to note that when dealing with a small pellet, suspending it in a large volume of medium can excessively dilute the cells, potentially hindering accurate quantification by a cell counter. If the volume needs to be reduced afterwards, additional centrifugation steps may be required, which could adversely affect cell vitality and viability. To prevent this, it is advisable to begin with a small volume of medium (e.g., 1 mL) to resuspend the pellet, increasing the volume gradually only if needed. Figure 2 illustrates representative cell pellet sizes after centrifugation and the recommended medium volumes for resuspension.*

6. Count the cells

a. Transfer equal volumes of cell suspension and Trypan Blue (10 μL + 10 μL, respectively) to an Eppendorf tube.

b. Collect 10 μL of the mixture and pipette it onto the slide for measurement, ensuring that no air bubbles form in the measuring window.

c. Place the slide in the cell counter and read the calculated number of viable cells per 1 mL of suspension.

*Note: Depending on the planned experiments and the number of cells required, seed the cells into a larger flask (150–300 cm^2^) and continue culturing until the desired cell number is achieved. Alternatively, if the number of cells is already sufficient, begin the ALI culture.*

**C. Establishing ALI culture**

1. Prepare materials and reagents

a. Prior to initiating the culture under ALI conditions, collect and count the cells following the procedure outlined above.

b. Determine the required number of inserts for the planned experiments, ensuring sufficient cell quantity, bearing in mind that each insert requires 0.1 × 10^6^ cells (final density insert directly is approximately 3.0 × 10^5^ cells/cm^2^).

c. Conduct all steps under sterile conditions within a biosafety cabinet. To disinfect the tweezers, transfer 5–10 mL of 70% ethanol into a 15 mL centrifuge tube and submerge them in it for 5–10 min.

d. Remove the tweezers and allow them to air dry. Avoid contact with any other equipment or surfaces to maintain sterility.

e. Prepare the required number of inserts and culture plates in advance. Open the seal and, using sterile tweezers, grasp one of the three designated handles to carefully transfer the insert into the wells of the culture plate.

*Note: Maintain strict aseptic technique throughout the process. Avoid placing inserts in edge wells; instead, fill those wells with 1 mL of sterile water to maintain high humidity and minimise the risk of cell desiccation. To enhance workflow efficiency, particularly when handling multiple plates with inserts, use a disinfected, lidded plastic container (plastic box) as a container for all plates.*

f. After completing the seeding procedure, place all plates inside the container, along with an open centrifuge tube containing 2–3 mL of sterile water, to help maintain humidity. Keep the container in the incubator with the lid slightly ajar.

2. Seed the cells in liquid–liquid culture

a. Pipette 0.1 × 10^6^ cells suspended in 400 μL of complete medium into the interior of each insert.

b. Add 1 mL of complete medium directly into the well beneath the insert to maintain liquid–liquid culture conditions for 7 days (at 37 °C in a humidified atmosphere with 5% CO_2_ and 20% O_2_).

c. Leave one insert unseeded and handle it identically to the others containing cells. This insert will serve as a blank control for TEER measurements.

d. Replace the medium in all inserts every 48 h: 400 μL in the insert and 1 mL in the well.

3. Establish ALI culture

a. After 7 days of liquid–liquid culture, carefully remove the culture medium from the wells and inserts (without damaging the cell layer in the insert).

b. Add 270 μL of culture medium to the well and incubate at 37 °C in a humidified atmosphere with 5% CO_2_ and 20% O_2_.

c. Change the medium every 48 h.

d. Place the plastic container holding all plates and the tube filled with sterile water in the incubator, ensuring the lid remains slightly open to maintain humidity.

*Note: The height of plates and inserts may vary slightly depending on the batch and/or manufacturer. Therefore, the volume of medium added beneath the insert under ALI conditions may need to be adjusted. The bottom of the insert should be slightly submerged in the culture medium. If, 24 h after adding 270 μL beneath the insert, medium accumulates inside the insert, carefully remove the excess. When replacing the medium, reduce the volume added beneath the insert by the amount previously collected from its interior. Carefully check for air bubbles under the insert.*

**Figure 3.**
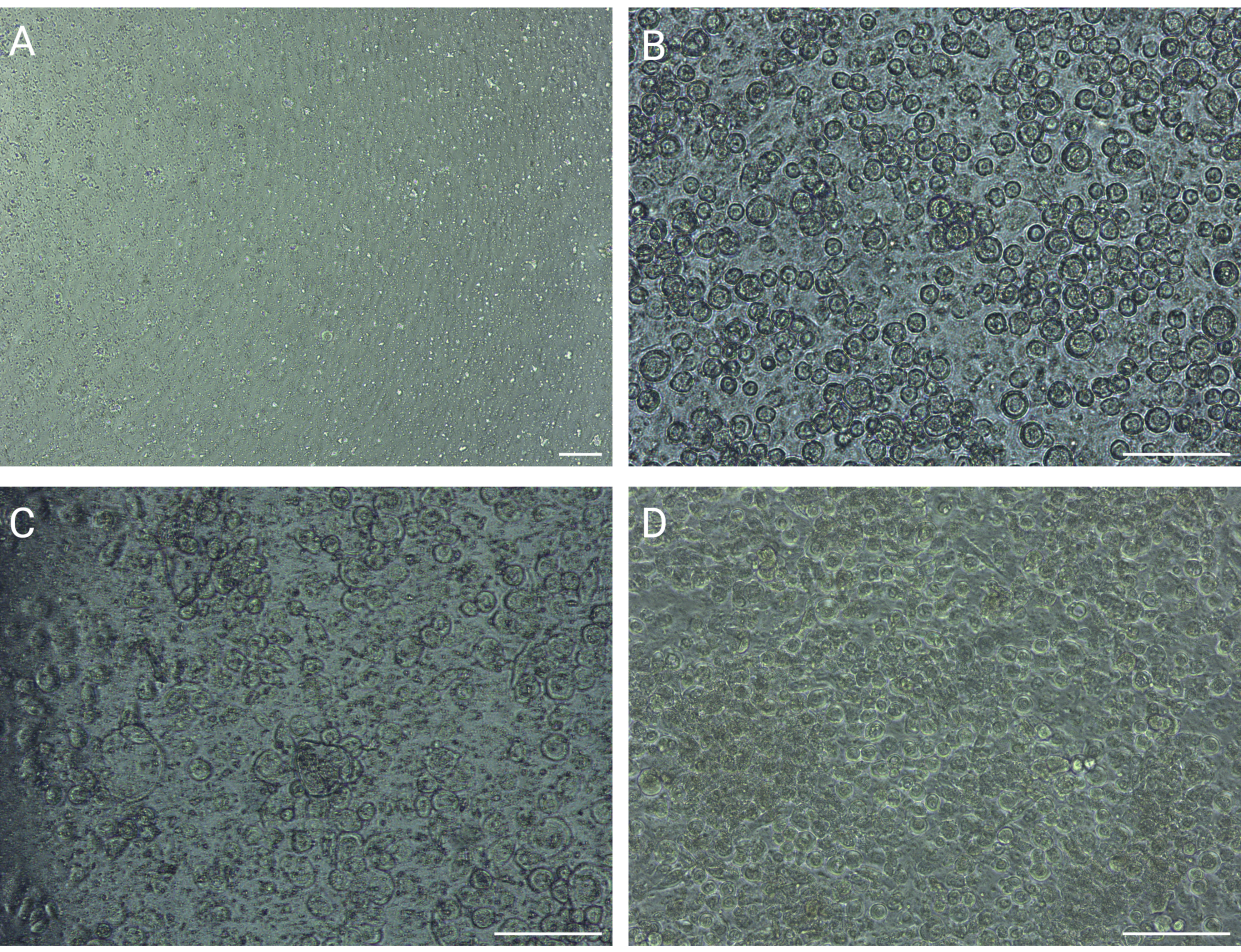
Cell morphology during culture on an insert membrane. (A) Empty membrane. (B) Cells after seeding on the insert. (C) First day of air–liquid interface (ALI) culture. (D) Twenty-first day of ALI culture. Scale bars, 100 µm. Magnification: 4× (A) and 10× (B–D).

4. Continue the ALI culture with cell integrity monitoring

a. Keep the plates in the incubator (37 °C, 5% CO_2_, 20% O_2_, humidified conditions).

b. Replace the medium beneath the inserts every 48 h (around 270 μL).

c. To monitor barrier integrity, measure TEER at least once a week, or more frequently if necessary. Figure 3 illustrates the morphology of HSAECs grown on inserts across different time points.

**D. TEER measurement**

1. Prepare materials and reagents

a. Prewarm the appropriate volume of complete medium in a water bath set to 37 °C.

*Note: Temperature plays a critical role in the accuracy of the readings: elevated temperatures increase medium conductance, leading to reduced resistance values, whereas a drop in temperature can transiently raise TEER. Consequently, any temperature fluctuation between measurements may introduce considerable variability in the recorded results. Therefore, standardisation of the procedure is crucial. To ensure consistency across all experiments and replicates, the culture medium temperature in both wells and inserts must be comparable between each TEER measurement.*

b. Disinfect the electrode by transferring 5–7 mL of 70% ethanol to a 15 mL centrifuge tube and submerging it for 2–3 min.

c. Remove the electrode from the ethanol and let it air dry completely.

d. Repeat the process using 5–7 mL of prewarmed complete medium to condition the electrode and allow it to air-dry as previously.

**Figure 4.**
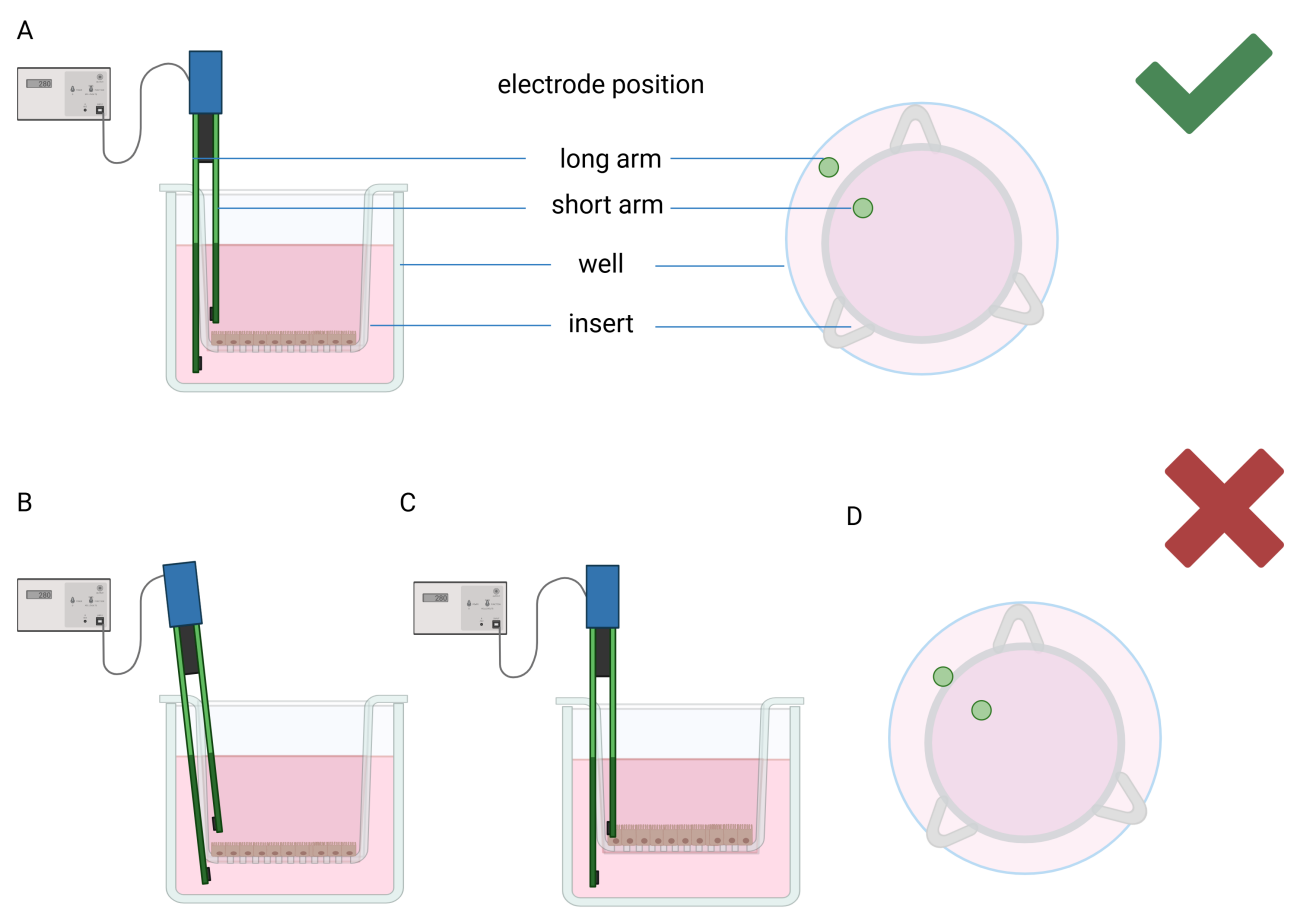
Sketch of electrode placement in the insert/culture plate well. (A) Correct position. (B–D) Examples of incorrect electrode position: (B) electrode held at an angle; (C) short electrode arm contacting the membrane/cell layer; (D) electrode contacting the insert walls.

*Note: During this process, avoid touching the electrode to maintain sterility. Note that the electrode height is adjustable. For accurate TEER measurements, both electrode arms must be fully immersed in the culture medium. The longer arm should gently reach the bottom of the well, while the shorter arm should not contact the insert membrane, as this could disrupt the cell layer. It is essential to determine the optimal electrode height experimentally before the first reading (e.g., in the blank well) and maintain that setting throughout all subsequent measurements. Figure 4 illustrates proper and improper electrode placement in the insert/well.*

e. Set up the Volt-Ohm Meter Millicell TEER measurement unit by powering on the device and inserting the test electrode to begin calibration. The display should read 1,000 Ω; if it does not, adjust the settings manually to match the correct value.

f. After calibration, plug in the measurement electrode and wait a few minutes for stabilisation; the unit is now ready to begin recording TEER values.

*Note: The operation of TEER measurement devices may vary depending on the manufacturer. If you are using equipment different from the one specified in this protocol, please follow the manufacturer's instructions for proper use and calibration.*

2. Replace the culture medium

a. Remove the culture medium from the well.

b. Add 400 μL to the insert and 1 mL to the well of the prewarmed culture medium and keep the plates in the incubator for 15 min to stabilise.

c. Place the plate outside the safety cabinet for an additional 15 min to allow the temperature to equalise.

*Note: These steps ensure that a similar measurement temperature is maintained for all measurements throughout the ALI culture.*

3. Measure the resistance

a. Begin by measuring the baseline resistance using the blank insert (without cells). Carefully immerse the electrode, ensuring the shorter arm is placed inside the insert and the longer arm in the well below.

*Note: Take care not to touch the edges of the insert and always position the electrode vertically at a 90° angle relative to the well surface. Allow a moment for the reading to stabilise; the resistance values may fluctuate initially but should settle within approximately 1 min.*

b. Once the resistance value is stable, write down the value obtained.

c. Proceed to measure the resistance in the remaining inserts using the same technique.

d. After completing the first round of measurements, repeat the process to have at least two independent readings per well. Use the average values to calculate the final resistance.

e. After finishing the measurement, clean the electrode by immersing it in 70% ethanol for 2–3 min and then allow it to air-dry.

4. Calculate the TEER value

a. The TEER (Ωcm^2^) formula is based on the NET resistance, which is the resistance (Ω) measured in cell-covered insert minus the resistance of a blank well and multiplied by the surface area of the insert (in cm^2^).

Example:

Value measured for blank insert: 280 Ω

Value measured for cells covered insert: 350 Ω

The information on the insert’s surface area (cm^2^) is available on the insert box’s specification label or on the manufacturer's webpage. For inserts suitable for a 24-well plate, the surface area is 0.33 cm^2^.

NET: 350 Ω - 280 Ω = 70 Ω

70 Ω × 0.33 cm^2^ = 23.1 Ωcm^2^

The TEER value for the tested insert is 23.1 Ωcm^2^.

## Data analysis

**Figure 5.**
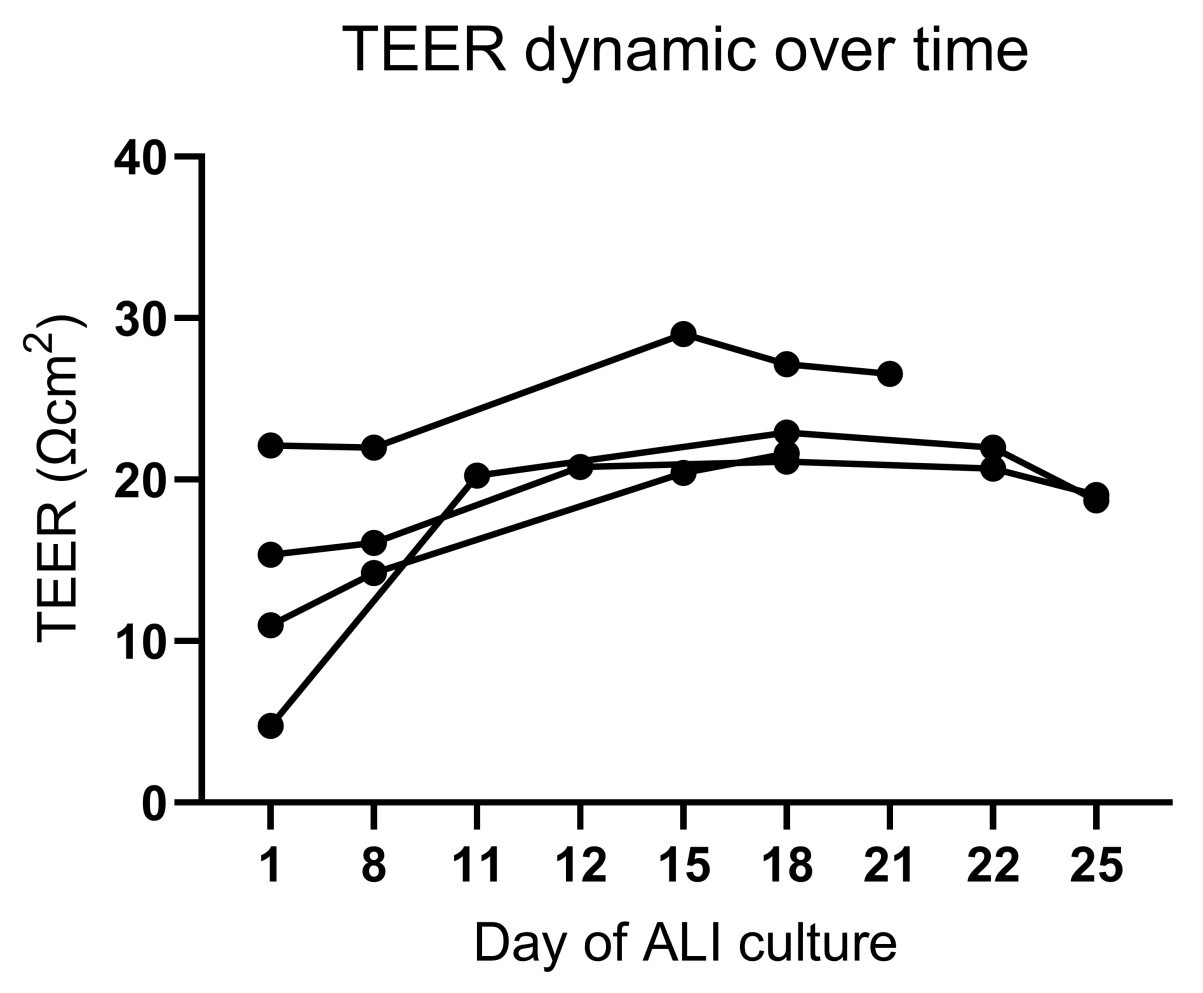
Transepithelial electrical resistance (TEER) dynamics over time across four biological replicates, starting from day 1 of air–liquid interface (ALI) culture

The cells were cultured according to the procedure described above, adapted from the manufacturer's guidelines [8] with minor modifications. An average of 10 inserts was seeded per culture plate, with plates 1–4 containing 10, 12, 10, and 8 inserts, respectively. Each plate also included one empty insert serving as a blank, processed identically to the seeded inserts but without cells. TEER measurements were performed alternately by two researchers, beginning on the first day of transition to ALI conditions. Individual TEER measurements for each insert were taken in duplicate and then averaged. The detailed data obtained and analysis methodology are provided in Supplementary Material 1. Table 1 presents the mean TEER values from four biological replicates and for each insert, compiled using Microsoft Excel. Descriptive statistical analysis and graphical presentation of TEER dynamics over time were performed with GraphPad Prism (Table 2 and Figure 5, respectively). The highest variability was observed on day 1, with discrepancies gradually decreasing over time. Between days 18 and 25, TEER values showed the greatest consistency across plates, indicating that this period represents the most stable phase of the culture and is optimal for downstream experimental applications. This observation aligns with previous reports that respiratory epithelial cells typically form a differentiated monolayer starting around day 21 of ALI culture [9].

**Table 1.** Individual values of transepithelial electrical resistance (TEER) measurements in time

Day of ALI culture	1	8	11	12	15	18	21	22	25
TEER value from individual inserts (Ωcm^2^)	14.025	14.52	20.79	20.46	20.295	21.285	27.555	17.985	19.14
	13.695	12.54	22.11	18.975	21.12	18.81	25.575	19.47	18.975
	15.51	16.005	20.295	17.325	22.44	20.295		20.46	16.83
	15.675	15.345	18.15	18.15	19.635	19.305		19.47	20.625
	15.18	18.645	20.13	23.595	20.955	20.13		20.13	
	16.005	18.315	20.13	25.08	19.14	20.295		21.78	
	13.86	8.58	21.12	17.82	20.79	23.76		19.305	
	17.16	19.305	20.46	25.245	22.44	24.585		24.915	
	14.355	20.13	20.955	21.945	20.955	19.635		20.955	
	18.15	17.49	18.315	19.14	16.335	23.265		22.44	
	-0.33	14.19	22.605		27.225	22.935		20.625	
	7.26	9.075	17.82		28.875	23.595		24.255	
	5.445	17.325			28.215	20.295		20.625	
	-11.55	12.045			33.66	20.955		22.605	
	9.24	15.18			32.505	22.935		21.285	
	4.29	13.365			26.895	18.48		19.965	
	8.085	11.55			27.39	21.615		20.955	
	5.445	14.19			27.39	23.265		21.615	
	3.96	16.83				27.39		23.595	
	9.405	18.645				25.41		22.275	
	9.405	21.285				23.265		21.78	
	6.435	20.955				24.915		24.09	
	-8.25	20.79				18.48			
	6.6	26.235				19.965			
	18.48	20.625				25.245			
	11.385	21.45				22.44			
	14.85	22.77				19.965			
	10.725	21.78				21.945			
	16.665					22.44			
	10.395					18.645			
	13.86					24.255			
	15.18					22.77			
	23.265					29.04			
	21.615					26.235			
	19.965					21.12			
	20.46					24.255			
	27.555					35.145			
	22.77					28.215			
	19.635					28.545			
	21.615					24.585			

**Table 2.** Descriptive statistics for transepithelial electrical resistance (TEER) values over time

Day of ALI culture	1	8	11	12	15	18	21	22	25
Number of values	40	28	12	10	18	40	2	22	4
Minimum	-11.55	8.58	17.82	17.33	16.34	18.48	25.58	17.99	16.83
25% percentile	7.466	14.19	18.77	18.07	20.67	20.3	25.58	20.09	17.37
Median	13.94	17.41	20.38	19.8	22.44	22.85	26.57	21.12	19.06
75% percentile	17.9	20.75	21.08	23.97	27.6	24.59	27.56	22.48	20.25
Maximum	27.56	26.24	22.61	25.25	33.66	35.15	27.56	24.92	20.63
Range	39.11	17.66	4.785	7.92	17.33	16.67	1.98	6.93	3.795
Mean	12.44	17.11	20.24	20.77	24.24	22.99	26.57	21.39	18.89
Std. deviation	8.044	4.282	1.498	3.007	4.882	3.438	1.4	1.771	1.562
Std. error of mean	1.272	0.8092	0.4324	0.9509	1.151	0.5435	0.99	0.3776	0.7812

## Validation of protocol

**Figure 6.**
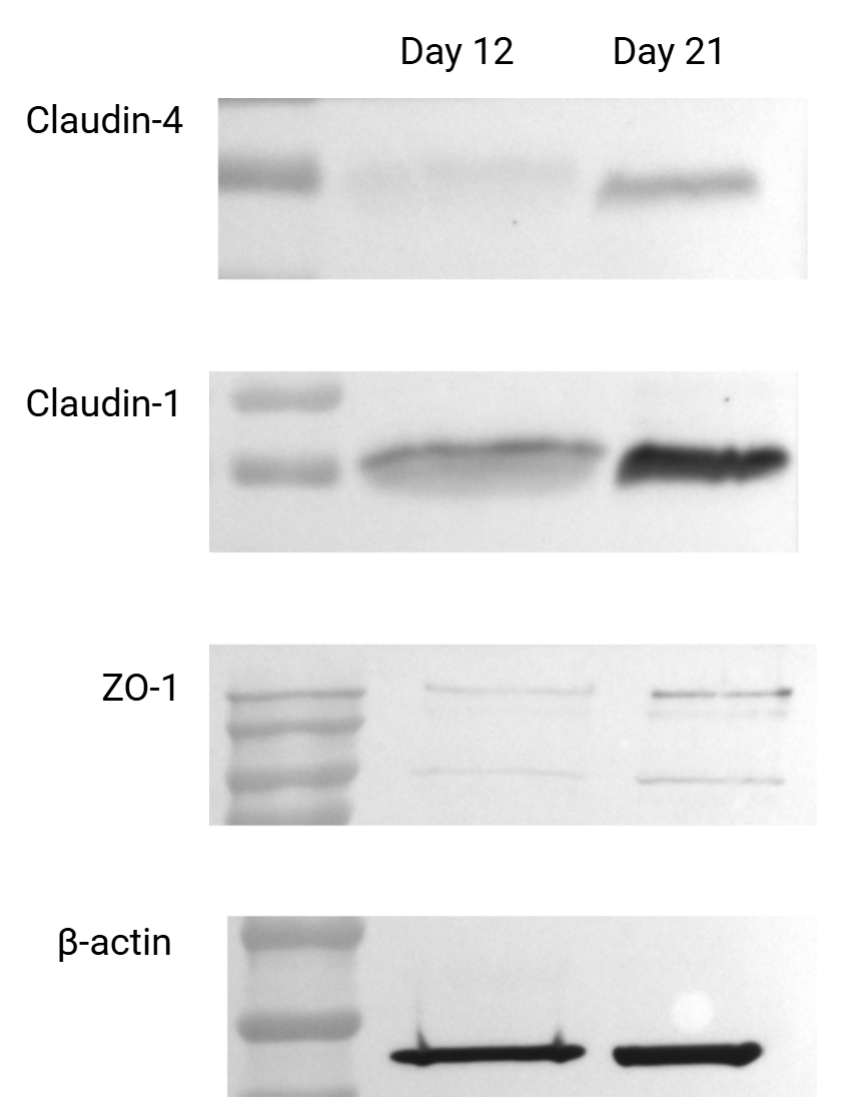
Visualisation of tight junction proteins (ZO-1, claudin-1, and claudin-4) in human small airway epithelial cell (HSAEC) cultures maintained under air–liquid interface (ALI) conditions for 12 and 21 days. Western blotting was performed using ZO-1, claudin-1, claudin-4, and β-actin antibodies at a 1:1,000 dilution, in accordance with established protocols [10].

Since TEER values are closely linked to tight junction expression, the presence of ZO-1, claudin-1, and claudin-4 was assessed on days 12 and 21 using western blot. Representative results are shown in Figure 6.

## General notes and troubleshooting

**General notes**

It is important to note that HSAECs are highly sensitive to storage conditions and gradually lose their physiological properties with increasing passage number. Upon receipt of the initial vial, it is advisable to expand the cells and prepare cryopreserved backups. Passage numbers beyond 5–6 are not recommended, as cellular functionality and differentiation capacity may be compromised. For ALI cultures, it is advisable to use cells from early passages and to avoid prolonged cryopreservation whenever possible, as extended freezing may compromise their functional integrity. Cell properties, including the ability to adhere, may also vary depending on the donor. Even when purchasing commercially available vials of primary cells, problems with cell adherence may occur. In that case, additional coating of the inserts, such as with collagen, may improve attachment and monolayer formation. However, if the researcher chooses to include a coating step, it should be applied consistently across all experimental samples to ensure a uniform environmental background.

**Troubleshooting**

**Problem 1:** Cells fail to adhere after seeding from a backup.

Possible cause: Cell damage due to prolonged storage or excessive passage number.

Solution: Use freshly thawed cells from an early passage.

**Problem 2:** Cells detach from the insert surface.

Possible causes: High passage number, reduced proliferative capacity, mechanical destruction, e.g., by tip scratching, too cold media, or low humidity.

Solutions: Use low-passage cells, minimise mechanical stress, prewarm media, and maintain high humidity by positioning inserts centrally in the plate, filling empty wells with sterile water, and placing an additional sterile water tube inside the incubation container.

**Problem 3:** Low TEER values.

Possible causes: Incomplete monolayer formation, reduced proliferative capacity, or barrier disruption due to mechanical damage.

Solutions: Allow an additional 7–14 days for recovery, then remeasure; if TEER remains unchanged, restart the culture with a new backup.

**Problem 4:** TEER values fluctuate across technical repetitions.

Possible cause: Inconsistent temperature of the culture medium during measurements.

Solution: Use media at a consistent temperature and standardise incubator and handling times to allow proper thermal equilibration.

## Supplementary information

The following supporting information can be downloaded here:

1. Supplementary Material 1. Results from individual TEER measurements, plates 1–4.
